# Combined JAK- and TNFα-targeting in severe steroid-refractory graft-versus-host disease in children

**DOI:** 10.1038/s41409-026-02926-w

**Published:** 2026-06-12

**Authors:** Manon Queudeville, Jaspar Kloehn, Ysabel Alessa Schwietzer, Kai Lehmberg, Johanna Schrum, Ingo Müller

**Affiliations:** Division of Pediatric Stem Cell Transplantation and Immunology, Clinic of Pediatric Hematology and Oncology, University Medical Center Hamburg-Eppendorf, Hamburg, Germany

**Keywords:** Graft-versus-host disease, Drug therapy

## Introduction

Steroid-refractory graft-versus-host disease (SR-GvHD) after hematopoietic stem cell transplantation (HSCT) remains a major cause of morbidity and mortality [1, 2]. The REACH trials demonstrated a better overall response and a better failure free survival in patients treated with the JAK inhibitor ruxolitinib for acute and chronic SR-GvHD compared to standard of care [3–5]. Of the 16 pediatric patients with higher grades acute GvHD (aGvHD) III–IV however, only 68.8% showed a response on day 28 while almost half of patients failed to maintain a durable response (ORR day 56, 56.3%) [5]. Other treatment options or combinations are needed for these patients.

Tumor necrosis factor alpha (TNFα) is a pro-inflammatory cytokine that is part of the initiation and effector phase of aGvHD [6]. We evaluated pediatric patients with severe SR-GvHD receiving a TNFα antagonist, JAK1/2 inhibition, or the combination of both.

## Methods

This study is a retrospective single center analysis over 12 years from January 2013 to December 2024. Thirty-seven patients (8.6%) out of 432 consecutive patients developed severe SR-GvHD. They were treated with anti-TNFα antibodies and/or ruxolitinib. Severe SR-GvHD included grades III and IV aGvHD as well as severe chronic GvHD (cGvHD). The median follow-up was 40 months (range 2–140 months). The definitions of GvHD grades and response as well as the drug dosages are outlined in the supplement.

## Results

Thirty-seven out of 432 (8.6%) pediatric patients transplanted during the 12 years developed severe SR-GvHD and were treated with anti-TNFα antibodies, ruxolitinib, or a combination thereof. Twenty-eight patients suffered severe SR aGvHD with a median time of onset of 33 days after HSCT and nine patients developed severe SR cGvHD with a median time of onset of 293 days after HSCT. The most commonly affected organ in patients with SR-aGvHD was the intestinal tract, the most common manifestations in SR-cGvHD were skin, joints and fasciae. Patient characteristics and GvHD manifestations are detailed in the supplementary information.

**Fig. 1:**
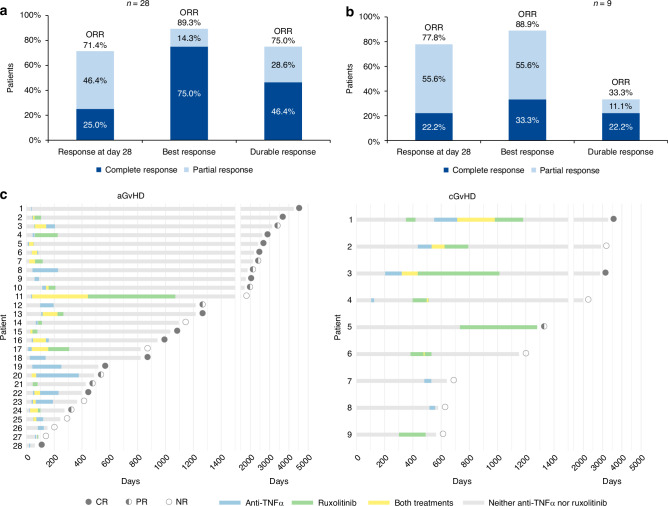
Response to treatment with ruxolitinib and/or anti-TNFα. **a** Response in patients with grades III or IV steroid-refractory acute graft-versus-host disease. **b** Response in patients with severe steroid-refractory chronic graft-versus-host disease. **c** Swimmer plot illustrating individual treatment timelines and durable responses in patients with acute and chronic GvHD. Each horizontal bar represents one patient (with corresponding patient ID) and depicts the treatment course from transplantation to last follow-up, plotted along the x-axis as days after transplantation. Patients with acute or chronic GvHD were treated with anti-TNFα therapy (infliximab or adalimumab); blue, ruxolitinib; green, or a combination of both; yellow. Treatment response is indicated by circular symbols and reflects the durable response achieved at the earliest time point, defined as the sustained clinical response during long-term follow-up. Filled circles denote complete response (CR), half-filled circles indicate partial response (PR), and open circles represent no response (NR).

Anti-TNFα treatment was administered in 91.9% of patients, while 78.4% received ruxolitinib. Most patients received both anti-TNFα and JAK inhibition (70.3%), either simultaneously or in succession. The overall response rate (ORR) at day 28 in grade III–IV SR-aGvHD was 71.4% and 75.0% of patients held durable responses (Fig. 1a). In SR-aGvHD, the best ORR at any given time point of treatment was 89.3%. The patients with SR-cGvHD also had a good response to treatment at day 28 with an ORR of 77.8%. The best ORR at any given time point of treatment of SR-cGvHD was 88.9%, but there were less patients achieving a complete response (CR) and more patients failing to maintain a durable response compared to severe SR-aGvHD. A durable response was achieved in 33.3% of SR-cGvHD patients (Fig. 1b). Taken together, 72.9% of the patients with severe SR-GvHD responded to treatment with TNFα inhibition and/or ruxolitinib at day 28 and 64.8% of patients also achieved durable responses. The swimmer plots in Fig. 1c display the exact individual treatment timelines for all patients up to last follow-up. The respective durable responses are specified.

Overall survival of the patients with SR-GvHD was 73.0% with only 8/37 (21.6%) cases of TRM, two patients died of relapse (5.4%). The GvHD free survival in this severely affected cohort of 37 patients at 40 months follow-up was 59.5%.

## Discussion

Treatment of severe SR-GvHD with ruxolitinib and/or TNFα-inhibition led to a meaningful clinical response in over 70% of patients at day 28 and two-thirds of patients showed durable response at last follow-up. The evaluation at last follow-up warrants a maximum observation period to detect durable effectiveness. Only eight patients out of 37 died due to TRM, the rate of death due to SR-GvHD and related complications in our cohort compares favorably with earlier reports [1, 2].

Ruxolitinib was a gamechanger in the treatment of SR GvHD and is now the standard second-line option after steroids, possibly moving to front-line soon. However, it has some limitations, such as severe cytopenias, especially thrombocytopenia [7, 8]. Here, TNFα-inhibition has the potential as a treatment not only for ruxolitinib-refractory patients, but for patients with severe ruxolitinib-induced adverse events.

Infliximab has been shown to be well tolerated and active for the treatment of steroid-resistant aGvHD in adults, particularly with gastrointestinal tract involvement, but a phase III study evaluating the addition of infliximab to methylprednisolone for patients with newly diagnosed aGvHD failed to show a benefit over corticosteroids alone [9].

Treatment with infliximab, initially once per week, for a median of eight doses in 24 children with SR-GvHD led to an ORR of 82% (12 CR and 6 PR) [10]. Achieving a CR or PR to infliximab was found to be the only factor predictive of prolonged survival. Infliximab showed a promising immediate effect, but often failed to maintain the treatment response. Some GvHD patients developed antibodies against infliximab despite extensive immunosuppression. Similarly, a prospective study in pediatric patients with aGvHD of liver or gut reported that infliximab as first-line treatment showed a markedly higher response rate than in second-line treatment [11].

The combination of ruxolitinib and etanercept, a TNFα receptor fusion protein, was studied in a multicenter prospective study with 64 adult and teenage patients with grades III–IV SR-aGvHD. The ORR was 87.5% at day 28 of the combination treatment and 73.4% reached a CR. A marked reduction of ≥75% in daily corticosteroid dosing was documented in 75.4% of patients at day 28. The 2-year OS after the combination therapy was 61.2% [12]. Comparable, or even more promising, effects for this type of combination treatment are shown in the study presented here in a relatively large pediatric cohort of severe SR-GvHD.

The limitation of this study is its retrospective, observational nature. Strengths are the relatively high number of children with severe SR-GvHD, the high level of data completeness, the lack of confounding and differing co-medication with other drugs against GvHD in the responding patients and the very long follow-up. Low absolute numbers of pediatric patients with SR-GvHD make prospective clinical trials extremely challenging. Therefore, such retrospective single-center analyses are of utmost importance to deliver first insights for new treatment combinations.

Our current analysis strongly advocates further investigation of combined JAK- and TNFα-targeting strategies in SR-GvHD. Trough levels of TNFα-targeting molecules and timely detection of drug antibodies may further improve rational dosage of available products. Anti-TNFα treatment will have to be evaluated alongside emerging new treatment options. Multicenter, and preferably multi-national, efforts will be necessary to conceive studies for the treatment of pediatric steroid- and ruxolitinib-refractory GvHD.

## Supplementary information

Supplemental Material

## Data Availability

The datasets are available from the corresponding author.
