# Supplementary material for: Combined JAK- and TNFα-targeting in severe steroid-refractory graft-versus-host disease in children
Source: Bone Marrow Transplant. 2026 Jun 12;61(8):1108–10. doi: 10.1038/s41409-026-02926-w (PMC13437246; doi:10.1038/s41409-026-02926-w)
Supplement: Supplementary file 1 [file 41409_2026_2926_MOESM1_ESM.docx]

**Supplementary information for the correspondence article:**

**Combined antagonisation of TNFα and JAK as treatment for severe steroid-refractory graft-versus-host disease in pediatric HSCT**

**Methods**

GvHD

Acute GvHD was graded based on the MAGIC criteria (1). If there was progression after 3 days or no improvement after 7 days on 2 mg/kg/d of methylprednisolone, the GvHD was declared as SR. The clinical grade of cGvHD was determined according to the modified criteria of the National Institutes of Health (2). SR cGvHD was defined as progressive disease after 1 to 2 weeks of treatment with methylprednisolone at ≥ 1 mg/kg/d or stable disease after 1 to 2 months on ≥ 0.5 mg/kg/d of methylprednisolone.

Evaluation of response

The treatment response was categorized as complete response (CR), partial response (PR), and no response (NR). CR required resolution of all symptoms related to GvHD. Improvement by at least one stage in the severity of GvHD in one organ without worsening in other organs was considered as PR. NR was defined as lack of improvement or progression of GvHD in at least one organ during the treatment. This included the occurrence of GvHD manifestations in a previously unaffected organ. Those who achieved a response due to additional agents beyond anti-TNF-alpha or ruxolitinib were also classified as NR.

The response rates to anti-TNFα, ruxolitinib, or their combination were assessed at different time points. The ORR comprises the proportion of patients with either CR or PR. We report ORR at day 28 of therapy, and durable response detected at the last follow-up. The first day of a durable response is defined as the day after which a patient did not experience further worsening of GvHD symptoms (for patients with PR) and for patients in CR the first day of durable response is the first day without a positive GvHD score and no further GvHD symptoms for the rest of the follow-up period. Furthermore, we analyzed overall survival, GvHD free survival and TRM.

Dosage of anti-TNFα and ruxolitinib

Infliximab was given intravenously at 5 mg/kg with an initial plan for 2 to 4 doses. Routinely, the first two doses were administered with an interval of one week. Further doses were administered depending on treatment response, with an interval of usually two weeks. In some patients, the dose was increased to 10 mg/kg.

Patients with an inadequate response to infliximab or adverse events related to infliximab received adalimumab subcutaneously. It was given as a biweekly standard dose of 20 mg (range 10 mg to 40 mg depending on patient's weight).

Ruxolitinib was administered orally and routinely started at a dose of 5 mg twice daily. Depending on the body weight, different dosages could be chosen. The average dose was 8.8 mg/m²/d. Dose adjustments were required in case of progressive GvHD or toxicity. Ruxolitinib was tapered based on persistent response. If an extension of the dosing interval to three times weekly was tolerated, ruxolitinib was discontinued.

**Suppl. Table 1: Baseline characteristics of patients and transplantation parameters.**

| **Characteristics** | **Number** |
| --- | --- |
| Total (%) | 37 (100) |
| Median age at HSCT, years (range) | 11 (0 – 16) |
| Gender (%)  Male  Female | 21 (56.8)  16 (43.2) |
| Diagnoses* (%)  Hematological malignancies  Hemoglobinopathies  Inborn errors of immunity  Inborn errors of metabolism  Bone marrow failure syndromes | 14 (37.8)  11 (29.7)  7 (18.9)  3 (8.1)  2 (5.4) |
| Donor type (%)  HLA-compatible unrelated   9/10 HLA-matched unrelated  HLA-identical related  HLA-haploidentical related | 19 (51.4)  11 (29.7)  6 (16.2)  1 (2.7) |
| Stem cell source (%)  Peripheral blood  Bone marrow | 25 (67.6)  12 (32.4) |
| GvHD prophylaxis (%)  Calcineurin inhibitors + mycophenolate mofetil  Calcineurin inhibitors + methotrexate  Calcineurin inhibitors + pt-cyclophosphamide  Calcineurin inhibitors  Others | 13 (35.1)  11 (29.7)  4 (10.8)  2 (5.4)  7 (18.9) |
| Acute GvHD (%)  Median time of onset, days pt | 28 (75.7)  33 |
| Chronic GvHD (%)  Median time of onset, days pt | 9 (24.3)  293 |
| Treatment with anti-TNFα (%)  Treatment with ruxolitinib (%) | 34 (91.9)  29 (78.4) |

^*^ Diagnoses were classified as hematological malignancies **(acute lymphoblastic leukemia,** acute myeloid leukemia, juvenile myelomonocytic leukemia, myelodysplastic syndrome), hemoglobinopathies (beta thalassemia major, sickle cell disease), inborn errors of immunity (leukocyte adhesion deficiency type 1, Griscelli syndrome type 2, chronic granulomatous disease, hemophagocytic lymphohistiocytosis), inborn errors of metabolism (mucopolysaccharidosis) and bone marrow failure syndromes (trilinear cytopenia of unknown cause, Diamond-Blackfan anemia).

GvHD graft-versus-host disease, HLA human leukocyte antigen, HSCT hematopoietic stem cell transplantation, pt post-transplant, TNF tumor necrosis factor.

**Suppl. Table 2 GvHD classification and affected organs.**

| **GvHD classification** | **Number of patients (%)** |
| --- | --- |
| **Acute SR-GvHD** | 28 (75.7) |
| Grade 3 | 11 (39.3) |
| Grade 4 | 17 (60.7) |
| **Acute SR-GvHD – organs involved** |  |
| Skin | 11 (39.3) |
| Gastrointestinal tract | 25 (89.2) |
| Liver | 8 (28.6) |
| **Chronic SR-GvHD** | 9 (24.3) |
| Severe | 9 (100) |
| **Chronic SR-GvHD – organs involved** |  |
| Skin | 8 (88.9) |
| Joints and fasciae | 7 (77.8) |
| Liver | 1 (11.1) |
| Lung | 3 (33.3) |

SR-GvHD steroid refractory graft versus host disease.
